# Progesterone Receptors in Prostate Cancer: Progesterone receptor B is the isoform associated with disease progression

**DOI:** 10.1038/s41598-018-29520-5

**Published:** 2018-07-27

**Authors:** Thea Grindstad, Elin Richardsen, Sigve Andersen, Kaja Skjefstad, Mehrdad Rakaee khanehkenari, Tom Donnem, Nora Ness, Yngve Nordby, Roy M. Bremnes, Samer Al-Saad, Lill-Tove Busund

**Affiliations:** 1 Department of Medical Biology, UiT The Arctic University of Norway, Tromso, Norway; 2 Department of Clinical Medicine, UiT The Arctic University of Norway, Tromso, Norway; 3 Department of Oncology, University Hospital of North Norway, Tromso, Norway; 4 Department of Clinical Pathology, University Hospital of North Norway, Tromso, Norway

## Abstract

The role of steroid hormones in carcinogenesis of the prostate is to some extent unraveled thorough the effect of androgen deprivation therapy on prostate cancer (PCa) progression. Other members of the steroid hormone family, such as progesterone, are also implicated in PCa, but progesterone’s role remains undefined. This study aimed to examine the distribution of progesterone receptor isoforms (PGRA, PGRB) in PCa tissue and their association with clinical endpoints. This was conducted retrospectively by collecting radical prostatectomy specimens from 535 patients. Tissue was analyzed using tissue microarray, where representative tumor areas were carefully selected. Protein expression was evaluated through immunohistochemistry, in stromal and epithelial tissue. Associations between receptor expression and clinical data were considered using statistical survival analyses. Herein, we discovered a solely stromal PGRA- and a stromal and epithelial PGRB expression. Further, a high PGRB expression in tumor tissue was associated with an unfavorable prognosis in both univariate and multivariate analyses: Biochemical failure (HR: 2.0, 95% CI: 1.45–2.76, p < 0.001) and clinical failure (HR: 2.5, 95% CI: 1.29–4.85, p = 0.006). These findings are in agreement with our previous investigation on pan-PGR, indicating that the observed negative effect of PGR is represented by PGRB.

## Introduction

With worldwide incidence- and mortality rates of estimated 1.600 000 cases and 366 000 deaths annually, prostate cancer (PCa) has been one of the most common cancers affecting males for decades^1^. Improved treatment strategies with drugs such as the new generation hormonal therapies, enzalutamide and abiraterone, has led to an increase in survival rates over the past years^2,3^. However, the nature of PCa remains a predicament for clinicians worldwide. The behavior of PCa has a broad specter, ranging from microscopic, well-differentiated tumors that remain indolent, to aggressive, high-grade tumors that eventually metastasize and result in morbidity and death. In addition, the heterogenous architecture of the tumors represent an impediment in the search for prognostic markers^4^. With limited progress in the development of prognostic markers, a great challenge remains in separating those in need of radical treatment from those with a disease that will never become clinically significant.

Steroid hormones constitute a large family of hormones, all originating from cholesterol. These hormones and their precursors are primarily synthesized and metabolized in the adrenal glands and gonads of men and women^5^. They exert their functions either by binding their respective receptors and thereby initiating specific receptor–protein- and receptor–DNA interactions, or as substrates for further metabolism to other steroid hormones^5^. In PCa, steroid hormones are considered tumor promoting factors. The proliferative effect has mainly been accredited to the androgens and the oncogenic role of the androgen receptor (AR) in tumor development, demonstrated by the effectiveness of androgen-deprivation therapy (ADT) on metastatic disease^6^. However, the PCa inevitably progresses despite of such treatment and becomes what today is known as “castration-resistant” prostate cancer (CRPC).

Progesterone is a steroid hormone which, in addition to being an intermediate step in the steroid hormone synthesis pathway, is well known for its important role in female reproductive organs^5^. Essential functions in male physiology have also been acknowledged^7^. Progesterone binds and stimulates the progesterone receptor (PGR) which exists in two isoforms, PGRA (94 kD) and PGRB (114 kD). Both receptors are transcribed from a single gene, separated only by additional 164 amino acids found in the upstream N-terminal region of PGRB. Despite these small differences, this region renders the PGRB with an extra activating function^8^ and evidence that the transcriptional activity of ligand bound PGRB is superior to that of PGRA has been presented^9^. Further, the isoforms are regulated by different estrogen receptor (ER)-inducible promotors and have their own response genes, mediating the wide spectrum of physiological effects of progesterone with little overlap^9,10^.

A role of the PGRs in tumorigenesis is now established in several malignancies. In breast cancer, PGR is regarded as a surrogate marker for ERα activity, due to the direct ERα mediated upregulation of the gene encoding PGR and the subsequent co-localization of the two receptors^11^. Its function in breast cancer development, however, remains unestablished, although the theory of an individual contribution by the PGR isoforms to malignant development is receiving attention^12,13^. Indeed, the presence of the PGRs has been confirmed in several other malignancies including endometrial cancer^14^, PCa^15–20^, lung cancer^21^ and astrocytomas^22^, although not necessarily separating between the two receptor isoforms. Altogether, this indicates the PGRs’ involvement in numerous biological processes throughout the human body and a broad spectrum of tissue specific receptor functions.

In a previous study, we described a negative effect on PCa outcome for patients with a high pan-PGR expression in tumor epithelial cells (TE). To further elucidate the significance of the PGRs in PCa, we systematically assessed both the stromal and epithelial expression of the two receptor isoforms, PGRA and PGRB, and evaluated their association with clinical outcomes in a large cohort of 535 PCa patients.

## Materials and Methods

### Patients and tissue data

This study includes tumor tissue and complete follow up data from 535 patients who underwent radical prostatectomy as initial PCa treatment. The material was collected retrospectively in the period 1995–2005 from the Departments of Pathology at the University Hospital of Northern Norway, Tromso (n = 248), St. Olav’s Hospital, Trondheim (n = 228), and Nordland Hospital, Bodo (n = 59). A total of 136 patients, of an original cohort of 671, were excluded from the study. Reasons for exclusion were: (1) radiotherapy to the pelvic region prior to surgery, (2) other malignancies within 5 years prior to the PCa diagnosis, (3) inadequate paraffin-embedded tissue blocks, (4) lack of clinical follow-up data or (5) due to hormonal therapy prior to or at the time of the prostatectomy. Demographic and clinical data were acquired from medical records. All prostate specimens were histologically re-evaluated and re-staged according to the 2010 TNM classification system^23,24^ by an experienced pathologist (ER). The tumors were further graded according to the modified Gleason grading system^25,26^. All patient data (Table 1) were registered in a SPSS data file and de-identified. Patient outcome data were collected until the last follow up date or patient death. Median follow-up time was 150 (range 18–245) months at the last patient update in December 2015. Detailed description of the cohort has been published previously^27^.

**Table 1:** Patient characteristics and clinicopathological variables in 535 prostate cancer patients (univariate analyses; log-rank test).

Characteristics	Patients		BF (n = 200, 37%)			CF (n = 56, 11%)		PCD (n = 18, 3%)	
	n	%	5 -year EFS (%)	10-year EFS (%)	p	10-year EFS (%)	p	10-year EFS (%)	p
**Age**					0.24		**0**.**038**		0.40
≤65	357	*67*	77	64		94		98	
>65	178	*33*	70	59		91		98	
**pT-stage**					**<0**.**001**		**<0**.**001**		**0**.**001**
pT2	374	*70*	83	73		97		99	
pT3a	114	*21*	61	45		87		98	
pT3b	47	*9*	43	22		74		90	
**pN-stage**					**<0**.**001**		**<0**.**001**		**<0**.**001**
NX	264	*49*	79	68		96		99	
N0	268	*50*	72	58		91		97	
N1	3	*1*	0	0		33		67	
**Preop**. **PSA**					**<0**.**001**		**0**.**029**		**0**.**003**
PSA ≤ 10	308	*57*	81	68		95		99	
PSA > 10	221	*42*	68	54		89		97	
Missing	6	*1*							
**Gleason grade group**					**<0**.**001**		**<0**.**001**		**<0**.**001**
1 (3 + 3)	183	*34*	83	70		98		99	
2 (3 + 4)	219	*41*	77	68		94		99	
3 (4 + 3)	81	*15*	70	47		90		96	
4 (4 + 4)	17	*3*	58	28		86		94	
5 (≥9)	35	*7*	37	29		65		91	
**Tumor size**					**<0**.**001**		**0**.**002**		0.09
≤20 mm	250	*47*	83	70		96		99	
>20 mm	285	*53*	68	55		90		97	
**PNI**					**<0**.**001**		**<0**.**001**		**<0**.**001**
No	401	*75*	80	70		96		99	
Yes	134	*25*	60	41		83		95	
**PSM**					**0**.**049**		0.20		0.84
No	249	*47*	80	66		96		98	
Yes	286	*53*	70	59		90		98	
**Circumferrent PSM**					**<0**.**001**		**<0**.**001**		**0**.**022**
No	381	*71*	82	70		96		99	
Yes	154	*29*	57	44		85		96	
**Apical PSM**					0.063		0.43		0.13
No	325	*61*	74	58		92		98	
Yes	210	*39*	77	68		93		99	
**LVI**					**<0**.**001**		**<0**.**001**		**<0**.**001**
No	492	*92*	77	64		95		99	
Yes	43	*8*	47	39		70		90	
**Surgical procedure**					0.47		0.31		0.96
Retropubic	435	*81*	77	63		92		98	
Perineal	100	*19*	68	58		95		99	

Significant p-values in bold (threshold p ≤ 0.05).

Patient characteristics and clinicopathological variables in 535 prostate cancer patients (univariate analyses; log-rank test). Significant p-values in bold (threshold p ≤ 0.05).

Abbreviations: EFS = Event free survival; BF = Biochemical failure; CF = Clinical failure; PCD = Prostate cancer death; PSA = Prostate specific antigen; PNI = Perineural infiltration; PSM = Positive surgical margin, LVI = Lymphovascular infiltration.

### Microarray construction

Tissue microarrays (TMAs) were constructed for the analysis of immunohistochemical (IHC) staining expression. For each case, a pathologist (ER) identified and marked representative areas of the prostate specimens with tumor epithelial cells (TE), tumor associated stromal cells (TS), normal epithelial cells (NE), normal stromal cells (NS), in addition to areas with benign prostate hyperplasia (H) and prostate intraepithelial neoplasia (PIN). From each of these areas, cores were sampled from each donor block in order to construct TMA blocks. Prostate cores from 20 patients without any history of malignancy were used as controls.

The TMAs were assembled using a tissue-arraying instrument (Beecher Instruments, Silver Springs, MD, USA). A 0.6 mm diameter needle was used to harvest cores from the marked tissue areas from the corresponding formalin-fixed paraffin-embedded tissue blocks. The samples were inserted into an empty recipient paraffin block according to a predefined coordinate pattern. To include all core samples, twelve tissue array blocks were constructed. Multiple 4 μm sections were cut with a Micron microtome (HM355S), affixed to glass slides. The detailed methodology has been previously reported^28^.

### Immunohistochemistry

**Figure 1:**
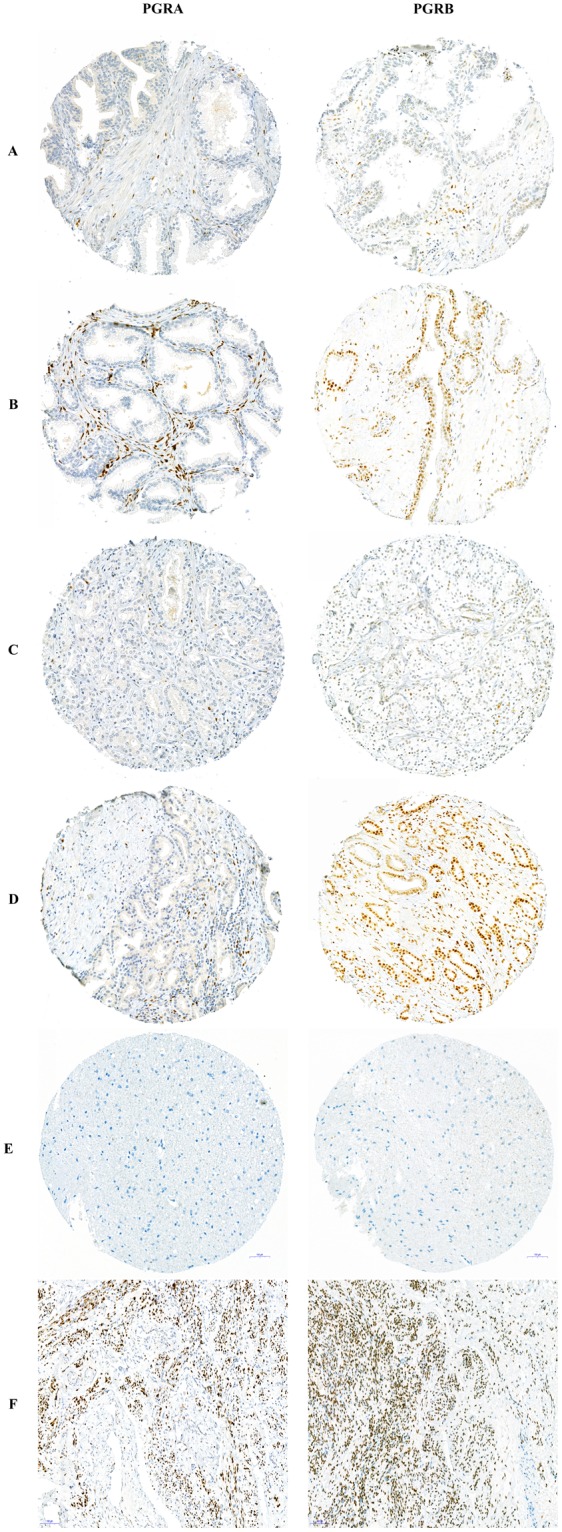
Immunohistochemical staining for progesterone receptor A and B. Representative pictures of immunohistochemical staining for progesterone receptor A and B (PGRA and PGRB) expression in tissue microarray cores from prostate cancer prostatectomy specimens in addition to positive and negative control tissue. Microscope pictures taken with 15x magnification. PGRA panel: (**A**) low and (**B**) high stromal PGRA expression in a normal tissue core, (**C**) low and (**D**) intermediate - high stromal PGRA expression in a tumor tissue core, (**E**) negative tissue control in normal human brain tissue (**F**) positive tissue control in normal human endometrial tissue. PGRB panel: (**A**) low epithelial PGRB expression and low – intermediate stromal PGRB expression in a normal tissue core, (**B**) high epithelial PGRB expression and intermediate – high stromal PGRB expression in normal tissue core, (**C**) low – intermediate epithelial PGRB expression and low stromal PGRB expression in tumor tissue core, (**D**) high epithelial and stromal PGRB expression in a tumor tissue core, (**E**) negative tissue control in normal human brain tissue (**F**) positive tissue control in normal human endometrial tissue.

The following primary antibodies were chosen in order to detect expression of PGRA and PGRB: Novocastra anti-human PGR (clone:16, cat # NCL-L-PGR-312) mouse monoclonal antibody, directed against the A isoform of the human PGR. An antibody acknowledged by the American Society of Clinical Oncology as a well validated antibody for evaluating the PGR in breast cancer using IHC^29^ (Supplementary Data 1). ThermoFisher anti-progesterone receptor (clone: hPRa2, cat # MA5-12642) mouse monoclonal antibody, directed against the B isoform of the PGR, validated by the manufacturer (Supplementary Data 2). We sought to validate the applied antibodies in-house prior to submissions. However, no PGR isoform-specific siRNA’s or transfected overexpressed lysates that could be used with the applied antibodies were commercially available. Additionally, the close molecular weight of the two receptor isoforms makes antibody validation challenging. According to the manufacturer (Novocastra) of the PGRA antibody, western blotting would detect both isoforms (PGRA and PGRB) and is thus not a suitable method for validation of the antibody. The manufacturer further describes that the reason IHC only detects the A isoform might be the result of the epitope being inaccessible in the folded B form of the PGR. To control the antibodies specificity, we performed staining of negative and positive tissue controls prior to the IHC procedures on the PCa cohort. Additionally, staining control was performed by removing the primary antibody during IHC procedure. Slides from multi-organ TMA blocks were used to verify staining specificity in each antibody optimization run. Samples from normal endometrium and normal brain tissue were included as positive and negative tissue control for PGRA and PGRB respectively (Fig. 1). Of note, an antibody directed against the PGRA isoform (cat # MA5-12658) from same company (ThermoFisher) as the applied PGRB antibody was initially attempted. However, the staining was unspecific and the experiment failed.

All TMA and control bocks required to be freshly sectioned for obtaining higher level of sensitivity. After overnight incubation of section slides at 60°, staining was performed with benchmark-ultra auto-immunostainer (Ventana). Slides were deparaffinized on the system with EZ Prep buffer for 3 cycles. A heat-induced pretreatment method was used in standard Cell Conditioning 1 (CC1) buffer at 95 °C with 64 (PGRA) and 48 (PGRB) min incubation time. The primary antibody was loaded at 1:25 (PGRA) and 1:50 (PGRB) dilution and was incubated for 60 min. The immune complexes were visualized with the optiView DAB Detection Kit (Ventana, # 760–700), followed by 4 (PGRA) and 8 (PGRB) minutes of amplification. Slides were counterstained with hematoxylin II (Ventana, # 790-2208) and bluing reagent (Ventana, #760-2037). Details regarding IHC control experiments are attached in the Supplementary File, Fig. 4.

### Scoring of Immunohistochemistry

All tissue samples were scored semi-quantitatively by two experienced investigators (ER, MR) independent of each other and blinded to any pathological or clinical information. The scoring was done manually using paired light microscopes. A third party (TG) recorded the mutely signaled values as the scoring progressed. In case of discrepancy (score difference > 1), the slides were re-examined and a consensus reached. Consequently, all reported marker expressions are based on two separate evaluations of the tissue cores.

Marker expressions were then evaluated in all different PCa compartments: NE, NS, H, PIN, TE and TS. Overall, the staining density of receptors displayed greater variation than the staining intensity, thus density was the chosen parameter. The density of PGRA and PGRB in each tissue compartment was given a score between 0–3, reflecting the percentage of positive cells in the examined compartment. The applied scoring system for both PGRA and PGRB is as listed: 0 = 0%, 1 = 1–25%, 2 = 26–50%, 3 = >50%. A core was scored as “missing” either if it was missing or considered of insufficient quality to score by both observers. There was a good scoring agreement between the two investigators (ER, MR) with a total intra-class correlation coefficient with absolute agreement (reliability coefficient, r) of 0.93 (95% CI: 0.92–0.94, p < 0.001). For each tissue compartment, the mean score was calculated and connected to the patient’s clinical and histopathological information. The scoring values were further dichotomized into low and high density. To secure reproducibility and after considering the p-value and patient distribution between the groups, the cut off was set at mean value: PGRA in TS ≤ 1,34, PGRB in TE ≤ 1,34, PGRB in TS ≤ 0,89.

### Statistical methods

All statistical analyses were performed with SPSS, version 24 (SPSS Inc., Chicago, IL, USA). The IHC scoring values from each pathologist were compared for inter-observer reliability by use of a two-way random effect model with absolute agreement definition. Correlation analyses were conducted using Spearman´s rank correlation coefficient to assess the correlation between the PGR´s expression, the clinicopathological variables and other previously published, potential prognostic markers. A correlation coefficient (r) of 0.3–0.49 was considered a moderate to weak correlation, r of 0.5–0.69 moderate to strong and finally r ≥ 0,7 as strong. In our material, only r > 0,3 was taken into consideration. The Wilcoxon signed ranks test was used to compare marker expression within the different PCa compartments. Univariate survival analysis was conducted using the Kaplan-Meier method with the log-rank test assessing the statistical significance between the survival curves of the model. The following end-points were considered in the survival analyses: (1) Biochemical failure (BF), (2) Clinical failure (CF) and (3) PCa death (PCD). BF was determined as prostate specific antigen (PSA) recurrence ≥0.4 ng/ml in a minimum of two different blood samples postoperatively^30^ and biochemical failure free survival (BFFS) was calculated from the date of surgery to the last follow up date for BF, which was the last date of a measured PSA. CF was defined as verified local symptomatic progression beyond cure and/or findings of metastases to bone, visceral organs or lymph nodes by CT, MR, bone scan or ultrasonography. Clinical failure free survival (CFFS) was calculated from the date of surgery to the last follow up date for CF, which was the last date without symptoms or any evidence of metastasis. PCD was defined as death caused by progressive and disseminated CRPC and prostate cancer death free survival (PCDFS) was calculated from the date of surgery to the date of death. All significant variables from the univariate analysis were entered in the multivariate analysis using a backward stepwise Cox regression model with a probability for stepwise entry removal at 0.05 and 0.1, respectively. We considered a p-value < 0.05 as statistically significant for all analyses. Presentations of the survival curves were terminated at 192 months due to less than 10% of patients at risk after this point.

### Ethics

This study was approved by the Regional Committee for Medical and Health Research Ethics, REK Nord, project application 2009/1393. A mandatory re-approval was conducted January 2016. As this was a retrospective study, where the majority of material was more than 10 years old, and most of the patients deceased, REK Nord considered written patient consent not necessary. All patients were made anonymous with each trial number. These numbers were initially linked to identity for only one purpose prior; to collect clinical information. The Data Protection Official for Research (NSD) approved the assembly of the database. The reporting of clinicopathological variables, survival data and biomarker expressions was conducted in accordance with the REMARK guidelines^31^.

### Data and material availability

A deidentified SPSS dataset including patient clinicopathological variables and marker expression variables is attached as a supplementary file.

## Results

### Patient characteristics

An overview of patient characteristics is presented in Table 1 and have previously been addressed in detail^27^. Median age at surgery was 62 years (range 47 to 76), the median PSA was 8.8 (range 0.7–104) and the median tumor size was 20 mm (2.0–50). The prostatectomy was retropubic in 435 cases (81%) and perineal in 100 cases (19%). Post-operative hormonal therapy was given to 89 (17%) of the patients and post-operative radiation therapy to 103 (19%), either due to rising PSA values, persisting PSA or unfree surgical margins. At the last follow-up in 2015, 200 patients (37%) had experienced BF, 56 (11%) CF and 18 (3%) had died due to PCa.

### PGRA and PGRB expression

PGRA expression was detected exclusively in stromal tissues and the staining was predominantly nuclear with a weaker cytoplasmic staining observed in some of the stained stromal cells. Expression of PGRB was located in both stromal and epithelial cells with a granular staining pattern in the nucleus. A weaker homogenous staining was also detected in the cytoplasm of a subgroup of both stromal and epithelial cells. The same expression patterns were also detected in the healthy control specimens. The stained stromal cells appeared morphologically to be mainly smooth muscle cells and fibroblasts. For both markers, the IHC staining was detected in a majority of tissue cores, this included both normal and tumor tissue compartments. Representative examples of PGRA and PGRB IHC staining in the PCa cohort are visualized in Fig. 1 and examples of PGRA and PGRB staining in TMA cores from healthy prostate tissue are visualized in Supplementary Fig. 3.

Of the 535 patients, 432 (81%) of the patients had TE and 454 patients (85%) TS that could be examined for PGRB and PGRA expression. Further, only 15 (3%) of the patients had a complete absence of stromal PGRA expression. Regarding PGRB, 96 (18%) of the patients had no epithelial expression and 102 (19%) patients had no stromal expression. A total of 69 (13%) patients had a combined negative stromal and epithelial expression of PGRB and 12 (2%) had neither PGRA nor PGRB expression. It was a significantly higher PGRA stromal cell density compared to PGRB in all stromal compartments (p < 0.001). As for PGRB, there was a significantly higher density of the receptor in epithelial tissue, compared to the surrounding stromal tissue (p < 0.001). Finally, no significant difference in density was detected between PGRA expression in TS compared to NS, nor to PGRB expression in TE or TS compared to NE and NS respectively. We did not detect any moderate or strong association between either PGRA nor PGRB and the clinicopathological variables listed in Table 1. There was a strong and significant correlation between PGRB in TE and TS (r = 0,82, p < 0,001), but no other significant correlation at a moderate or strong level was detected between the investigated markers and other previously published markers.

### Univariate analysis

**Figure 2:**
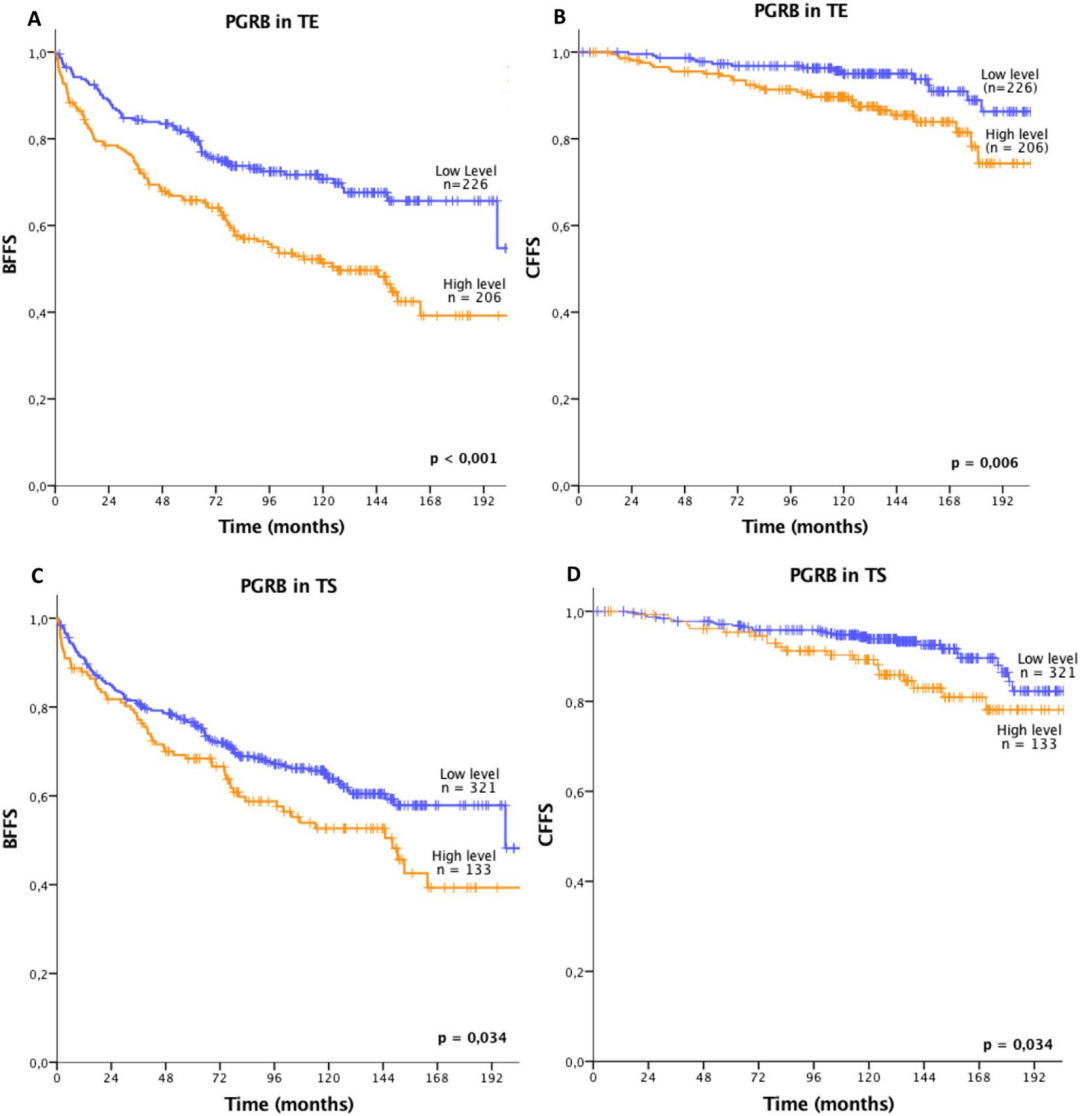
Kaplan-Meier curves presenting significant results from univariate analyses. The Kaplan-Meier curves demonstrate a high and low progesterone receptor B (PGRB) expression level dichotomized at mean value and the association with patient outcome. A reduction in biochemical failure free survival (BFFS) and clinical failure free survival (CFFS) was demonstrated for patients with a high expression of PGRB in both tumor epithelial cells (TE) **(A,B)** and tumor associated stromal cells (TS) **(C,D)**. Significant p-value in bold (threshold p ≤ 0.05).

Results from univariate analyses of clinicopathological variables and molecular markers and their association to the outcome measures (BF, CF, PCD) are presented in Tables 1, 2 and Fig. 2. A significant decrease in both BFFS and CFFS was observed for patients with a high PGRB expression in TE (BFFS: p < 0.001, CFFS: p = 0.006) and TS (BFFS: p = 0.034, CFFS: p = 0.034). No additional prognostic value was evident when merging PGRB expression in TE and TS. There was no significant association detected between PGRA expression levels in stromal cells and outcome measures (Supplementary Fig. 1). The same trend was observed when considering the results throughout the different pathological centers, however without significant levels for each subgroup (Supplementary Fig. 2).

**Table 2:** Significant results from univariate analyses of PGRB.

Marker expression	Patients		BF				CF		
	n	%	Events (n)	5-year EFS (%)	10-year EFS (%)	p	Events (n)	10 – year EFS (%)	p
**PGRB TE**						**<0**.**001**			**0**.**006**
Low	226	*42*	65	82	71		15	95	
High	206	*39*	99	66	51		30	90	
Missing	103	*19*							
**PGRB TS**						**0**.**034**			**0**.**034**
Low	321	*60*	133	77	64		27	94	
High	133	*25*	61	64	53		21	89	
Missing	81	*15*							

Expression of progesterone receptor B (PGRB) in tumor epithelial cells (TE) and tumor associated stromal cells (TS) of prostate cancer and its relation to clinical endpoints. The table presents the significant reduction in event-free survival (EFS) time for patients with high levels of PGRB in TE or TS (univariate analyses; log-rank test). Significant p-values in bold (threshold p ≤ 0.05).

Abbreviations: BF = Biochemical failure; CF = Clinical failure.

### Multivariate analysis

Multivariate analyses are presented in Table 3. When assessed alongside with the clinicopathological variables, a high PGRB expression in TE remained an independent prognostic marker for both BF (HR: 2.0, 95% CI: 1.45–2.76, p < 0.001) and CF (HR: 2.5, 95% CI: 1.29–4.85, p = 0.006). Regarding BF, a positive circumferential surgical margin, PNI, Gleason grade group (GGG) 3 (3 + 4) and 4 (4 + 3), preoperative PSA, and pT-stage 3b were additional independent prognosticators. Regarding CF, high PGRB expression in TE remained an independent marker alongside with age ≥60, LVI and Gleason grade group 1 through 5. PGRB in TS did not reach statistical significance in multivariate analyses.

**Table 3:** Results from multivariate analyzes.

Patient characteristics	BF			CF		
	HR	CI (95%)	p	HR	CI (95%)	p
**Age**	NE					**0**.**026**
≤65				1.0		
>65				2.0	1.10–3.80	
**pT-stage**			**0**.**004**	NS		
pT2						
pT3a	1.4	0.93–2.10	0.105			
pT3b	2.3	1.40–3.83	0.001			
**Preop PSA**			**0**.**021**	NS		
PSA ≤ 10	1.0					
PSA > 10	1.5	1.06–2.07				
Missing						
**Gleason grade group**			0.058			**0**.**013**
1 (3 + 3)	1.0			1.0		
2 (3 + 4)	1.3	0.87–1.95	0.203	3.3	1.01–10.01	**0**.**035**
3 (4 + 3)	1.7	1.05–2.75	**0**.**032**	5.8	1.80–18.50	**0**.**003**
4 (4 + 4)	2.7	1.30–5.50	**0**.**008**	6.3	1.37–29.00	**0**.**018**
5 (>9)	1.6	0.90–2.10	0.148	7.9	2.28–27.44	**0**.**001**
**Tumor size**	NS			NS		
≤20 mm						
>20 mm						
**PNI**			**0**.**002**	NS		
No	1.0					
Yes	1.7	1.22–2.45				
**Circumferrent PSM**			**0**.**016**	NS		
No	1.0					
Yes	1.5	1.10–2.10				
**LVI**	NS					**0**.**028**
No				1.0		
Yes				2.5	1.10–5.56	
**PGRB in TE**			**<0**.**001**			**0**.**006**
Low	1.0			1.0		
High	2.0	1.45–2.76		2.5	1.29–4.85	

Results from Cox regression analysis (backward stepwise model) displaying progesterone receptor B and the other remaining independent prognosticators for patient outcome in prostate cancer patients (n = 535), significant p-values in bold (threshold p ≤ 0.05).

Abbreviations: PGRB = Progesterone receptor B; BF = Biochemical failure; CF = Clinical failure; HR = Hazard ratio; CI = Confidence interval; PSA = Prostate specific antigen; PNI = Perineural infiltration; PSM = Positive surgical margin, LVI = Lymphovascular infiltration; TE = Tumor epithelial cells; NE = Not entered; NS = Not significant.

## Discussion

Herein, we demonstrated a wide distribution of the PGR proteins in both stromal and epithelial PCa tissues, which is in agreement with our previous report^18^. In this study, both isoforms of the PGR, PGRA and PGRB, where assessed. In our cohort, the PGRA expression was exclusive to stromal tissue, whereas PGRB expression was observed in both stromal and epithelial tissues. Further, we identified a significant decrease in BFFS and CFFS for patients with a high level of PGRB in TE with as much as 2.5 times increase in risk of CF. No such associations were observed for the PGRA. Hence, it is likely to assume that our previously observed impact of PGR expression in TE was indeed effectuated by the PGRB isoform^18^.

To our knowledge, Yu *et al*. is the only research group^15,32^ that has recently investigated PGR and its isoforms in PCa, including the various tissue compartments. Whereas our study detected PGRB expression in 81% (432 of 523 patients) of TE from the investigated patients, conflicting data regarding epithelial expression has been presented by Yu *et al*.^15^, detecting PGRB only in a subset of stromal cells in a small cohort of 27 prostatectomy cases. In their more recent work with IHC on TMAs from a larger cohort (n = 194), using a pan-PGR antibody, a great distribution of stromal PGR was described. The epithelial distribution of PGR was however not addressed^33^ and thus difficult to compare with our work. Using cell line studies, Yu *et al*.^33,34^ also demonstrated a favorable role of both PGR isoforms in regulating the stromal environment. This is, however, in contrast with our results where high PGRB levels in TS was associated with a worse prognosis in univariate analyses. This was however not statistically significant in multivariate analyses.

Earlier results supporting our observation of a negative role of the PGR in PCa have been published^16,17^, yet several previous publications are also in disagreement regarding PGR tissue expression, though most do not differ between the isoforms. Results regarding PGR’s presence in stromal tissue appears univocal^15–20^. The epithelial PGR distribution is however debated. While a total absence has been described by some groups^15,19^, other groups in addition to ours, clearly detect its presence^16–18,20^. Thus, the PGRs physiological function in the normal prostate and their role in PCa development is not yet defined. Interestingly, a selection of commercially available PGR specific antibodies have been compared in an earlier paper, in which a great variance in receptor expression was observed between the different antibodies^35^. All applied antibodies detected PGRA, but many failed to recognize PGRB in formalin fixed tissue. Moreover, the PGRA specificity of our applied antibody is supported by these investigations. These discrepancies may explain why some previous studies failed to recognize PGR in epithelial cells.

PGRA and PGRB have to a greater extent been investigated in female reproductive organs than the prostate, as outlined in the review by Scarpin and colleagues^36^. Herein, the observation by Mote *et al*.^12,37^ of a 1:1 receptor ratio of PGRA/PGRB in healthy female reproductive tissue is described and it is hypothesized that the majority of progesterone targeting tissue in humans have an expression profile not deviating far from this. A disruption of this receptor homogeneity has been demonstrated in different cancers. Mote *et al*.^12^ observed a PGRA predominance in human breast cancer lesions compared to the 1:1 ratio in healthy breast tissue. However, in endometrial cancer, the loss of equilibrium in PGRA/PGRB ratio and the subsequent predominance of either of the isoforms was observed as an early event in tumorigenesis^14^. In our material, there is also evidence of receptor disequilibrium as presented in the results section. In brief, this indicates that a receptor expression imbalance would result in changes in progesterone signaling in hormone-dependent tissues. This is supported by a recent study by Singhal *et al*.^13^ demonstrating that breast cancer tumors expressing higher levels of either PGRA or PGRB had different gene expression profiles and inhabited the ability to reprogram ER signaling in an independent manner. It would be of great interest to further assess the PGR isoform ratio in this prostate cancer cohort. However, considering the semi-quantitative scoring system applied herein, this is not a sufficiently precise method for assessing receptor ratio expression. A more appropriate approach for the future would be to assess the ratio after a full digital quantification of positive cells. Other groups have also applied immunofluorescence for the visualization of co-localization and ratio-assessment^12,37^, which is also an approach to consider for the future.

Several reflections must be made when considering how a high PGRB expression level can have a negative prognostic effect in PCa. Co-expression of steroid hormone receptors in hormone dependent cancers is prevalent, and recent discoveries have implicated a considerable interaction between these receptors. This could be either through regulation of receptors acting as cancer drivers or by oncogenic conversion of the receptors it self^13,38–41^. In PCa, the glucocorticoid receptor (GR) has been associated with tumor progression and enzalutamide resistance by reactivation of a selection of AR-target genes^38,39^. The PGR is, like the GR, similar to AR with a high sequence homology in the DNA-binding domain^42^, indicating a transferability of this theory to the PGRB. Results from breast cancer models indicate that the PGRs can modulate ER function and target gene activity through several mechanism, one being modulation of chromatin binding^40,41^. Similar mechanisms of interplay may exist between the PGRs and other steroid hormone receptors in PCa, but this warrants further investigation. Coregulatory proteins influence the expression and function of steroid hormone receptors. Aberrant expression of coregulatory proteins belonging to the p160 steroid receptor coactivator (SRC) family that are associated with modulation of the PGRs, such as SRC-2 and SCR-3, have been implicated in PCa and other hormone dependent malignancies^43,44^.

In summary, it is highly likely that our observed negative prognostic effect of a high PGRB expression in PCa is just the tip of the iceberg in a complex steroid hormone interplay in PCa development. To this date, the prognostic and therapeutic value of PGRA and PGRB in PCa remains undefined. However, the lack of available prognostic biomarkers, in addition to the progression of PCa to CRPC despite the emerging strategies targeting steroid hormones, makes this a subject for further investigation. Mifepristone is a compound with antagonistic abilities towards PGRs, AR in addition to the GR^45^. So far, the inhibitory effect of mifepristone on the GR in CRPC has been explored by Isikbay *et al*. reporting inhibition of CRPC growth and delayed progression in pre-clinical models^39^. This effect was however not observed in a small phase II clinical trial^46^. Alas, none of these studies considered expression of the PGR and its isoforms. Studies considering PGR inhibition in earlier stages of PCa is also lacking. There is however an ongoing phase I/II clinical trial investigating the effect of the anti-progestin onapristone in patients with CRPC and confirmed PGR expression^47^. This study may shed more light into this issue.

Major strengths of this study are our large, multicenter cohort (n = 535), the long follow-up time (mean 12.4 years), our precise and separate focus on stromal and epithelial tissue compartments in addition to our attempt to standardize cut-off values. However, this study approach has its natural limitations given that it only investigates associations between specific protein-expressions in PCa tissue and patient outcomes. It explains little about the underlying mechanisms regarding a potential oncogenic role of PGRB in PCa. Hence, at this stage, it can only be considered exploratory and hypothesis generating. Consequently, this study would greatly benefit from additional functional studies investigating the migratory and invasive potential of PGRB expressing cells. Another valuable approach for the future would be the investigation of the transcriptome regulated by the PGR isoforms in both epithelial and stromal cells, and also different cellular processes regulated by the different PGRs. Other natural limitations in this study are due to the nature of PCa and the subsequent low number of events (CF, and PCD) despite the long follow-up in our cohort that challenges the statistical analyses.

## Conclusion

Herein, we present the distribution of PGRA and PGRB expression in stromal and epithelial PCa tissue in a large cohort (n = 535) of primary PCas. We depict how PGRB in TE emerge as a strong independent predictor of PCa recurrence. No association with clinical endpoints was discovered for PGRA. This indicates that differences in PGR isoform expression may provide tumors with distinctive prognostic and hormone-responsive features, underscoring the importance of isoform specific evaluation of the tumors PGR status. It also raises the question whether treatment strategies targeting specific PGR isoforms in PCa might be beneficial. However, due to conflicting results in the current literature, further exploration is essential before the clinical value of the PGRB status is resolved.

## Electronic supplementary material

Supplementary information
