# Supplementary material for: Progesterone Receptors in Prostate Cancer: Progesterone receptor B is the isoform associated with disease progression
Source: Sci Rep. 2018 Jul 27;8:11358. doi: 10.1038/s41598-018-29520-5 (PMC6063894; doi:10.1038/s41598-018-29520-5)
Supplement: Supplementary file 1 — Supplementary information [file 41598_2018_29520_MOESM1_ESM.pdf]

Supplementary information

Progesterone Receptors in Prostate Cancer:

Progesterone receptor B is the isoform associated with disease progression

Thea Grindstad MD[1], Elin Richardsen, MD, PhD[1,4], Sigve Andersen MD, PhD[2,3], Kaja Skjefstad MD[1], Mehrdad Rakaee khanehkenari M.Sc[1], Tom Donnem, MD, PhD[2,3], Nora Ness MD[1], Yngve Nordby MD[2], Roy M. Bremnes, MD, PhD[1,2], Samer Al-Saad MD, PhD[1,4] & Lill-Tove Busund, MD, PhD[1,4]

1Dept. of Medical Biology, UiT The Arctic University of Norway, Tromso, Norway

2Dept. of Clinical Medicine, UiT The Arctic University of Norway, Tromso, Norway

3Dept. of Oncology, University Hospital of North Norway, Tromso, Norway

4Dept. of Clinical Pathology, University Hospital of North Norway, Tromso, Norway

Corresponding author and reprints:

Thea Grindstad

IMB - Dept. of Medical Biology

UiT The Arctic University of Norway

9019 Tromso, Norway

Telephone +47 95878050

Fax: +47 77672704

E-mail: tgr015@post.uit.no

Supplementary Figure 1.

Kaplan-Meier curves displaying results from univariate analyses of progesterone receptor A (PGRA) expression in prostate cancer tumor associated stroma (TS) and its association with biochemical free survival (BFFS) and clinical failure free (CFFS) survival. P-values in bold (significance threshold $p \leq 0.05$)

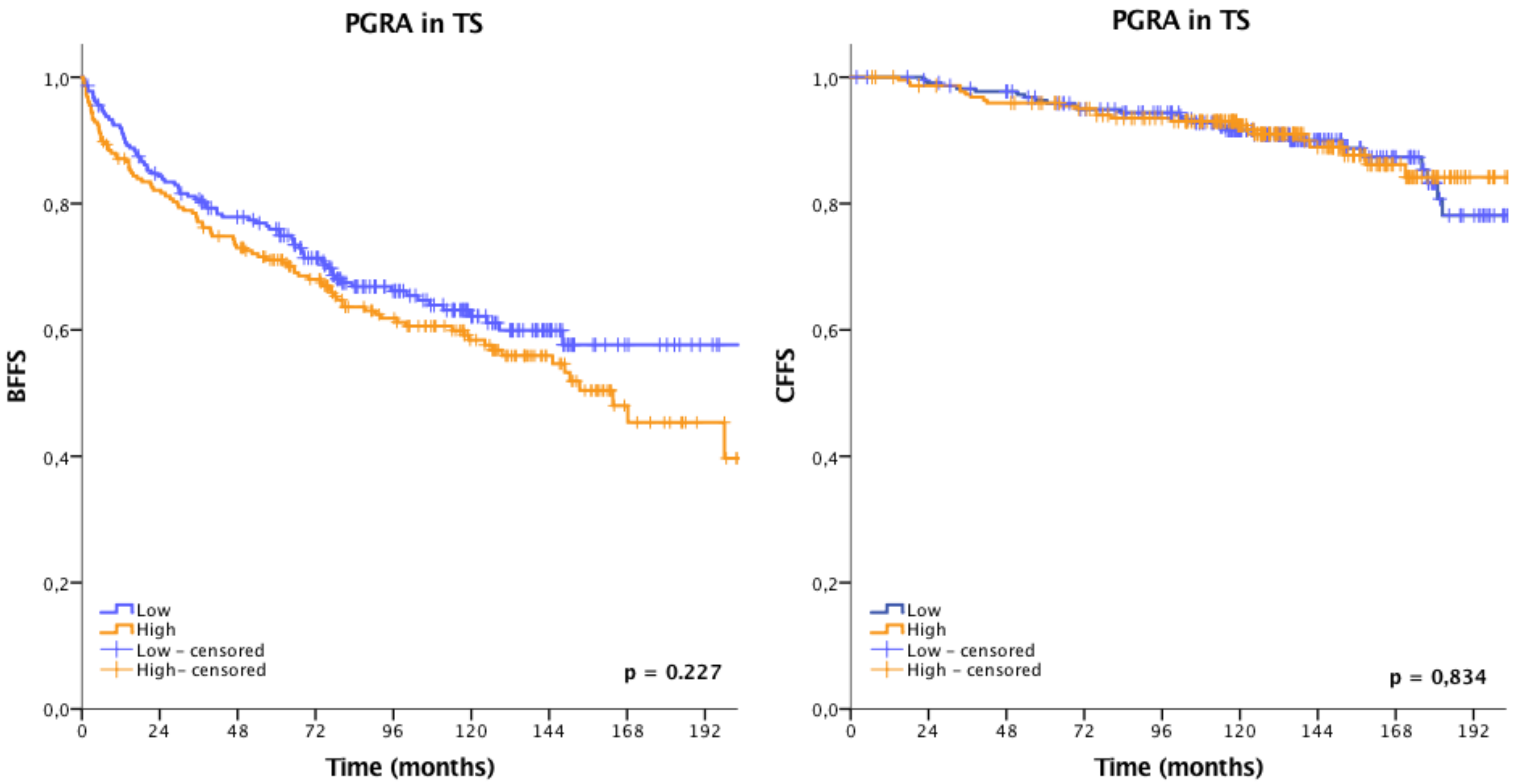

Supplementary Figure 2. Results stratified by pathological centers

Kaplan-Meier curves and table presenting results from univariate analyzes of progesterone receptor B (PGRB) expression in prostate cancer tumor epithelial cells (TE) stratified by the different pathological centers: University Hospital of Northern Norway, Nordland Hospital and St. Olav`s hospital. Biochemical Failure Free Survival (BFFS) is the presented outcome measurement. The same trends as presented in the main result were observed throughout the pathological centers, however without significant levels for each subgroup.

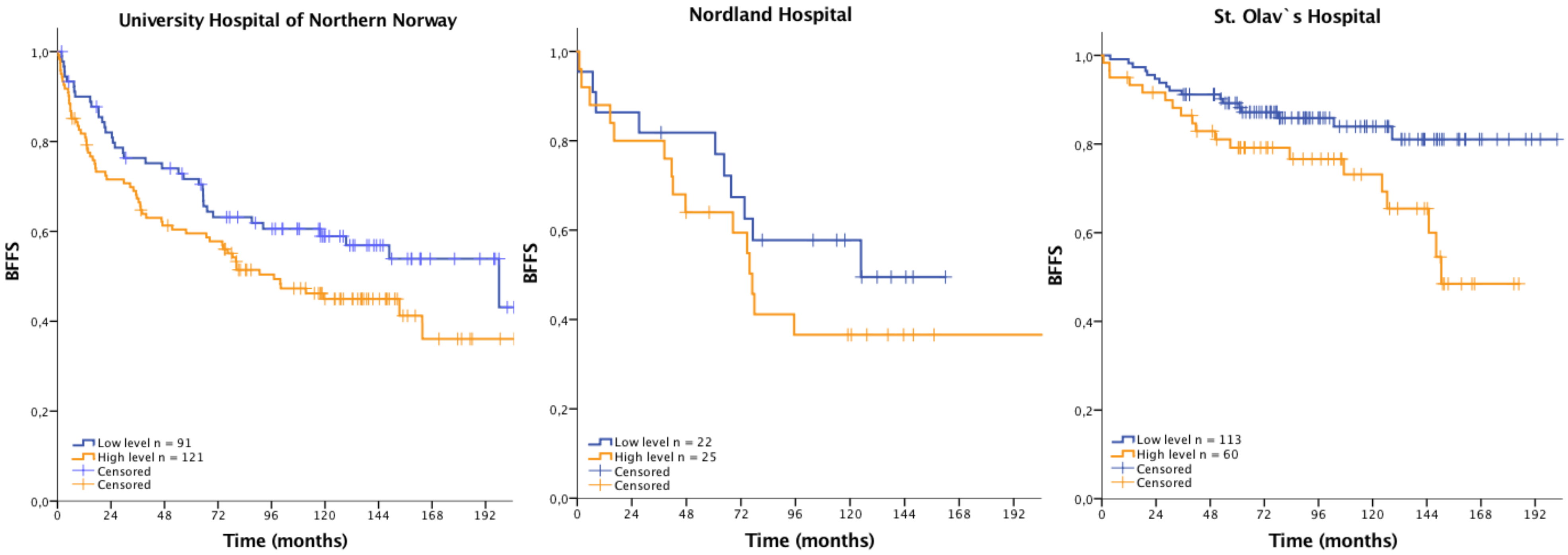

Pathological center	Number of patients (%)	p
University Hospital of Northern Norway	212 (49%)	0,060
Nordland Hospital	47 (11%)	0,306
St Olav`s Hospital	173 (40%)	0,015

Supplementary Figure 3. Expression of progesterone receptor A and B in healthy prostate tissue

Representative pictures of positive and negative immunohistochemical staining of progesterone receptor A and B (PGRA and PGRB) in tissue microarray cores from healthy prostate control tissue. PGRA staining was solely stromal and PGRB staining was both stromal and epithelial, resembling that of the prostate cancer cohort. **PGRA-panel: A)** negative PGRA expression, **B)** positive stromal PGRA expression. **PGRB-panel: A)** negative PGRB expression, **B)** positive epithelial- and stromal PGRB expression

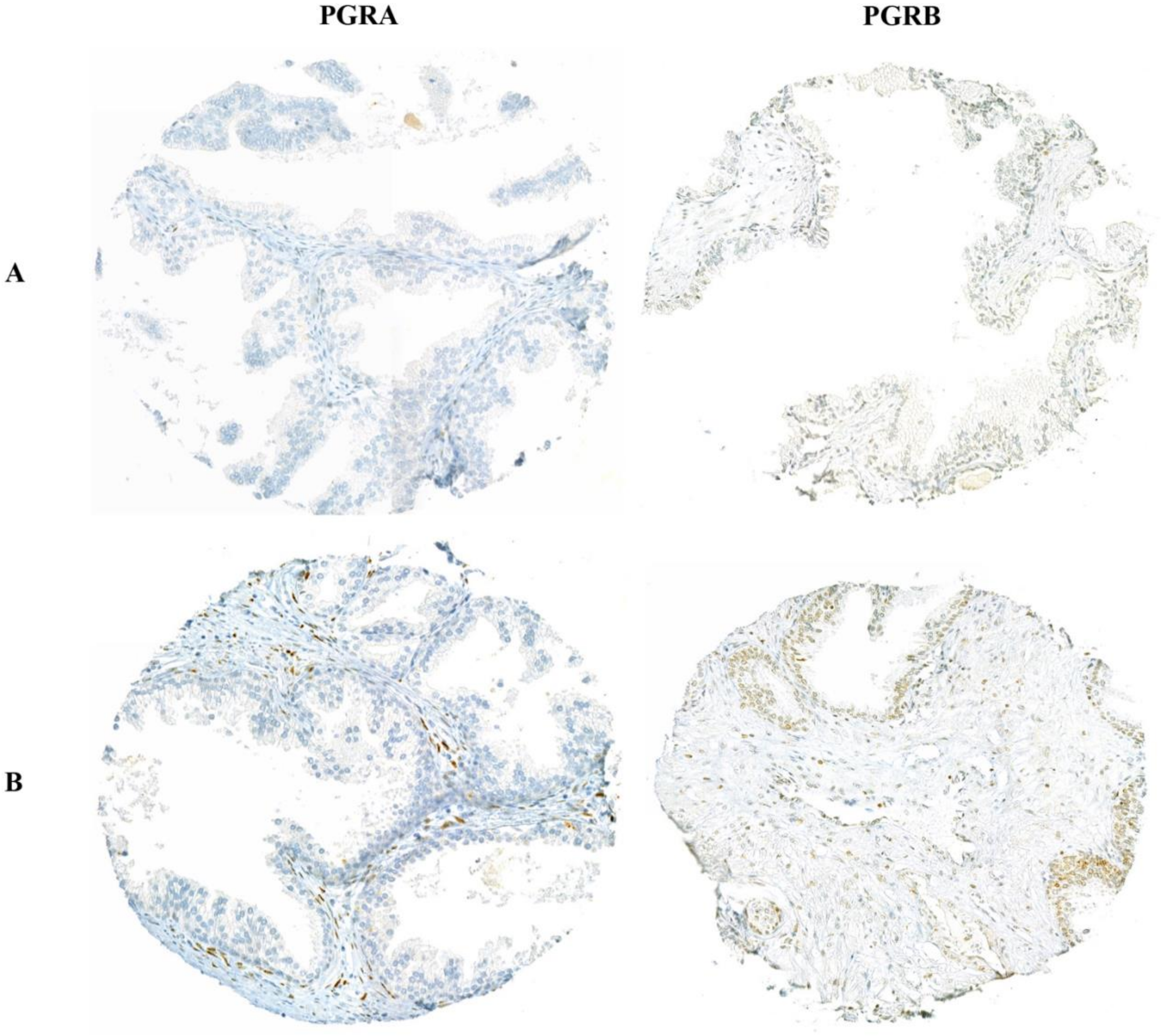

Supplementary Figure 4. Immunohistochemistry staining specificity and -quality on normal endometrial tissue control

Analysis of progesterone receptor A (PGRA) and progesterone receptor B (PGRB) staining specificity and -quality on normal endometrium at different baking temperatures (37°C and 60°C) and with different storing status (fresh and paraffin-coated stored section slides). A, B) Freshly sectioned slides for PGRA (A) and PGRB (B) staining after overnight incubation of slides in 37°C; C, D) Freshly sectioned slides for PGRA (C) and PGRB (D) staining after overnight incubation of slides at 60°C, demonstrating a similar staining intensity and -pattern in the control tissue at both baking temperatures (37°C vs 60°C). E, F) Three-month-old paraffin coated slides stained for PGRA (E) and PGRB (F) after overnight incubation of slides at 60°C, highlighting the importance of freshly used sections slides for these antigens. All slides are from the same case and the immunohistochemistry (IHC) procedure is in accordance with the IHC protocol and antibody dilution used for the main prostate cancer cohort (magnification x5).

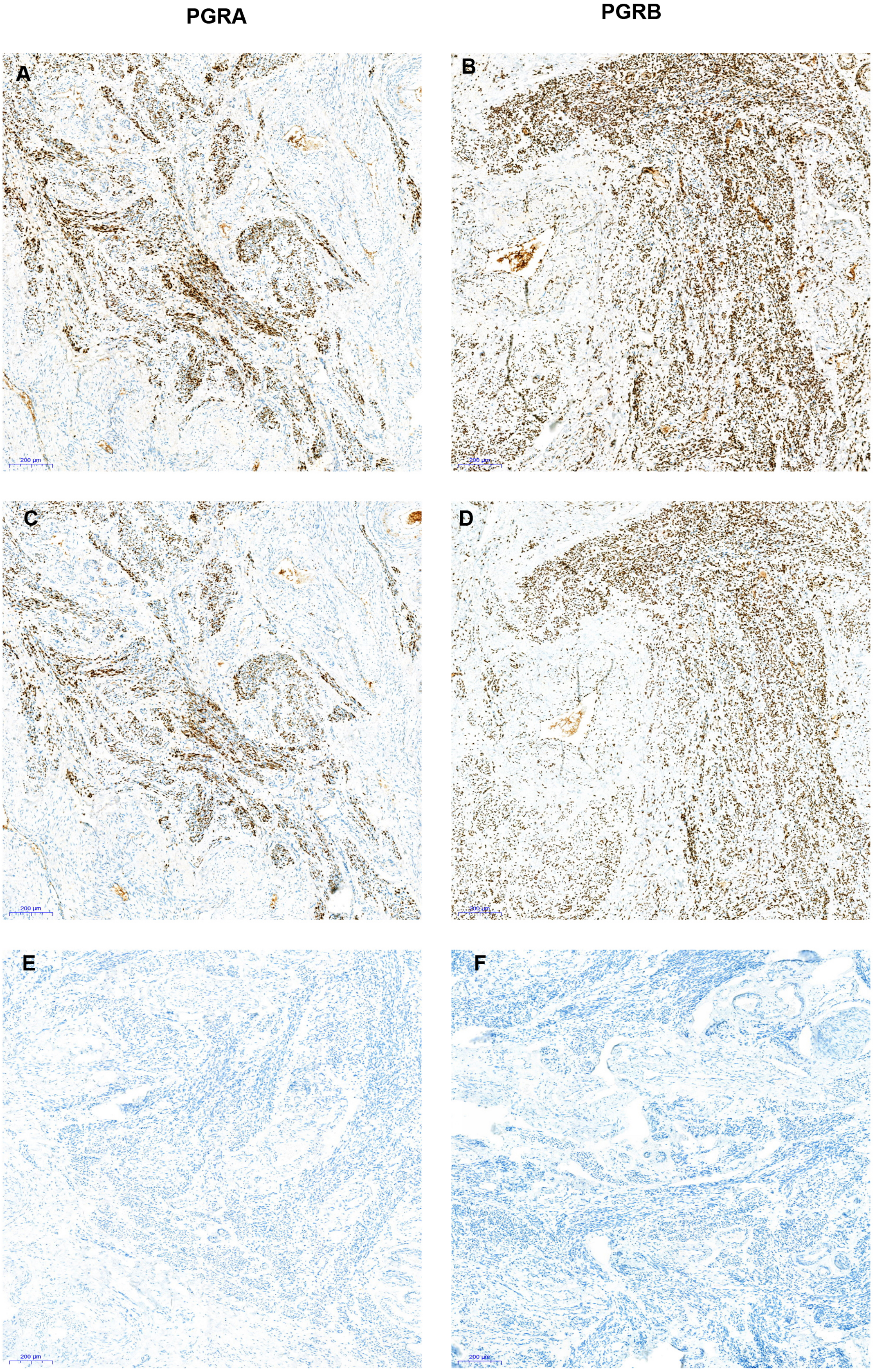
PGRA
PGRB
A
B
C
D
E
F
200 μm
